# The Structure and First-Passage Properties of Generalized Weighted Koch Networks

**DOI:** 10.3390/e24030409

**Published:** 2022-03-15

**Authors:** Jing Su, Mingjun Zhang, Bing Yao

**Affiliations:** 1 School of Electronics Engineering and Computer Science, Peking University, Beijing 100871, China; jingsu@pku.edu.cn; 2 Key Laboratory of High Confidence Software Technologies, Peking University, Beijing 100871, China; 3 China Northwest Center of Financial Research, Lanzhou University of Finance and Economics, Lanzhou 730020, China; 4 School of Information Engineering, Lanzhou University of Finance and Economics, Lanzhou 730020, China; 5 Key Laboratory of E-Business Technology and Application, Lanzhou 730020, China; 6 College of Mathematics and Statistics, Northwest Normal University, Lanzhou 730070, China; yybb918@163.com

**Keywords:** Koch network, degree distribution, diameter, random walk, average trapping time

## Abstract

Characterizing the topology and random walk of a random network is difficult because the connections in the network are uncertain. We propose a class of the generalized weighted Koch network by replacing the triangles in the traditional Koch network with a graph $R_{s}$ according to probability 0≤p≤1 and assign weight to the network. Then, we determine the range of several indicators that can characterize the topological properties of generalized weighted Koch networks by examining the two models under extreme conditions, p=0 and p=1, including average degree, degree distribution, clustering coefficient, diameter, and average weighted shortest path. In addition, we give a lower bound on the average trapping time (ATT) in the trapping problem of generalized weighted Koch networks and also reveal the linear, super-linear, and sub-linear relationships between ATT and the number of nodes in the network.

## 1. Introduction

Complex networks are acknowledged as an invaluable system for describing nature and society [1,2]; many endeavors have been devoted to exploring the structure and properties of complex networks for characterizing and simulating the properties of some real-world systems in our life. Among all of these properties, the scale-free nature, diameter, and clustering coefficient have attracted considerable attention [3,4,5]. In addition, the weight of the network has important research significance in air transportation [6], biological neural networks [7] and so on. Therefore, it is necessary to explore the influence of weight on the topological properties and dynamic process of the network, so we also determine the bound of the average weighted shortest path of network designed in this paper.

To better understand the properties of random networks in complex networks, extensive technical methods were developed for establishing a variety of theoretical models of random networks. For example, the well-known ER-model was proposed by Erdos and Renyi [8] to try to explain a low clustering coefficient and low variation in the node degrees; its degree distribution was verified to be Poisson distribution. Watts and Strogatz put forward a small-world WS-model [9], which can rationally reflect the statistical properties of the network that are neither completely regular nor entirely random and explain small-world phenomena in various real-world networks by exploring the diameter and clustering coefficient. The BA model was built by Barabasi et al. [10] using two rules, growth and preferential attachment; the degree distribution of the latter two networks obeys a power–law distribution. In this paper, we introduce a class of generalized weighted Koch networks with probability *p*. The ranges of their topological parameters are given by characterizing the topological characteristics of the deterministic network models under the two extreme states.

Random walk as a fundamental tool to describe the dynamic process of networks, such as page search in the world wide web [11], signal propagation [12] and energy transport [13]. The trapping problem is defined as a kind of random walk that takes place in networks in the presence of a fixed trap, absorbing all particles that visit it [14,15]. A basic quantity relevant to the trapping problem is called the mean first-passage time (MFPT). The MFPT from a node *i* to the trap is the expected time taken by a walker starting from *i* to reach the trap for the first time. The average trapping time (ATT) is the average of MFPTs over all starting nodes other than the trap. The ATT for a given trap is used as a indicator of the trapping efficiency to evaluate the process of trapping; it has a core position in many disciplines, including computer, biology, engineering and so on [16,17,18].

The organization of this paper is as follows. In Section 2, we introduce a method about constructs the generalized weighted Koch networks, according to probability *p*. In Section 3, we characterize several parameters that reveal the topological properties and dynamic processes of our network, including average degree, degree distribution, clustering coefficient, diameter, average weighted shortest path, and average trapping time. In the last section, we draw the conclusion with a concise narrative.

## 2. The Generalized Weighted Koch Network

The generalized weighted Koch network $G_{s,r}\left(t\right)$ presented in this paper is controlled by three parameters s≥3,r>0,t≥0, where *r* is the weight factor and *t* is the time step, and *s* and *t* are positive integers. We mainly explore the influence of the parameter *s* and the weight *r* related to the topology on the topological properties and dynamic characteristics of the network.

**Figure 1:**
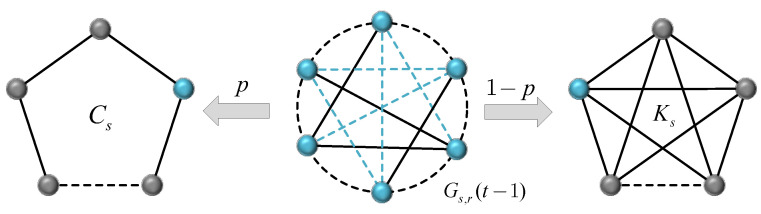
Iterative rule of the generalized weighted Koch network.

Let $C_{s}$ and $K_{s}$ be a cycle with *s* nodes and a fully connected graph with *s* nodes, respectively. We introduce a probability 0≤p≤1, then $G_{s,r}\left(t\right)$ can be created in the following way: for t=0, the network starts with a graph $R_{s}$ of *s* nodes, and its edge with unit weight corresponds to $G_{s,r}\left(0\right)$. When p=0, $R_{s}$ is a cycle $C_{s}$ of *s* nodes, and when p=1, $R_{s}$ is a complete graph $K_{s}$ of *s* nodes. For t≥1, $G_{s,r}\left(t\right)$ is obtained by adding a node group $R_{s}$ for each node in every existing graph $R_{s}$ of $G_{s,r}\left(t-1\right)$, where each node group $R_{s}$ can be a cycle $C_{s}$ with probability *p* or a complete graph $K_{s}$ with complementary probability 1−p; this rule is shown in Figure 1. We repeat this growth process until the network becomes what we need.

Additionally, for the generalized weighted Koch network we proposed, if r=1 and s=3 are satisfied, we can obtain the classic Koch network mentioned in the literature [19]; when r=1, we can obtain the expanded Koch networks referred to in Ref. [20]. For s=3, the network $G_{s,r}\left(t\right)$ is simplified to the weighted Koch network in Ref. [21]. The above network models are all special cases of the generalized weighted Koch networks constructed in this paper.

**Figure 2:**
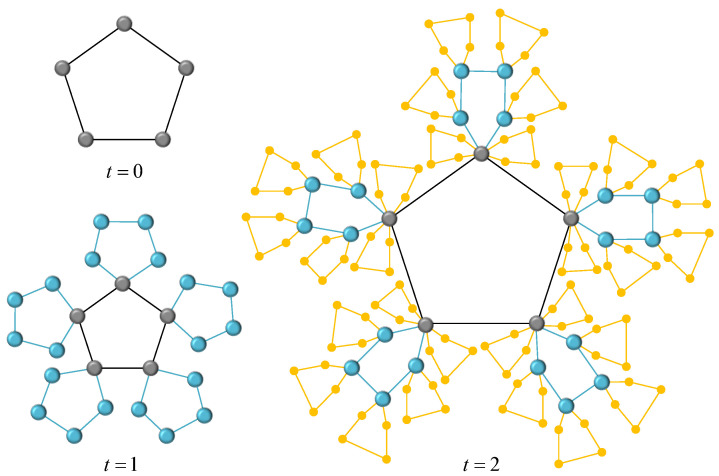
The network $G_{s,r}^{A}\left(t\right)$ at first three time steps when s=5 and r=1.

**Figure 3:**
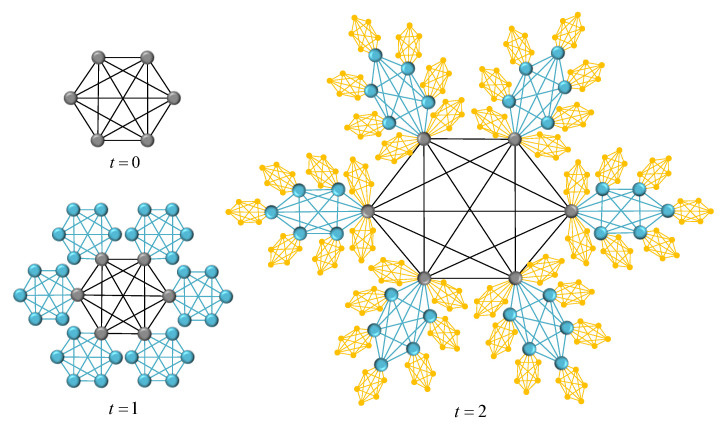
The network $G_{s,r}^{B}\left(t\right)$ at first three time steps when s=6 and r=1.

That is to say, $G_{s,r}\left(t\right)$ can be obtained from $G_{s,r}\left(t-1\right)$ by the recursive method, where one can connect each node of existing cluster $R_{s}$ in $G_{s,r}\left(t-1\right)$ with a graph of *s* nodes according to probability 0≤p≤1. We denote the two networks corresponding to the extreme conditions p=0 and p=1 as $G_{s,r}^{A}\left(t\right)$ and $G_{s,r}^{B}\left(t\right)$, respectively. Figure 2 and Figure 3 show the growing process of the two determined networks at t=0,1,2. Some topological properties and ATT of $G_{s,r}^{A}\left(t\right)$ have been described in the literature [22,23], so we mainly focus on the topological properties and ATT of $G_{s,r}^{B}\left(t\right)$, then estimate the range of parameters of random network $G_{s,r}\left(t\right)$ by studying the characteristics of two deterministic networks $G_{s,r}^{A}\left(t\right)$ and $G_{s,r}^{B}\left(t\right)$.

Let $N_{t}$ and $E_{t}$ be the number of nodes and edges of network $G_{s,r}\left(t\right)$, and let ΔN(t) and ΔE(t) be the number of new nodes and new edges created at time step *t*, that is $N_{t}=N_{t-1}+\Delta N\left(t\right)$ and $E_{t}=E_{t-1}+\Delta E\left(t\right)$. Additionally, $C_{s}$ or $K_{s}$ in Figure 1 is regarded as a cluster $R_{s}$, the total number of new generated clusters in the $G_{s,r}\left(t\right)$ at time step *t* is recorded as R(t), and combining R(t)=(s+1)R(t−1) and the initial value R(0)=1, we have $R\left(t\right)={\left(s+1\right)}^{t}$. Based on the construction method, we obtain

(1) $$\Delta N\left(t\right)=s\left(s-1\right)\cdot C\left(t-1\right)=s\left(s-1\right){\left(s+1\right)}^{t-1}.$$

The number of nodes and edges of networks $G_{s,r}^{A}\left(t\right)$ and $G_{s,r}^{B}\left(t\right)$ are denoted by $N_{t}^{Z}$ and $E_{t}^{Z}$, Z=A,B. The difference between $G_{s,r}^{A}\left(t\right)$ and $G_{s,r}^{B}\left(t\right)$ is that the cluster $R_{s}$ selected by the probability *p* is not the same, the number of nodes of all $R_{s}$ is *s*, but the number of their edges is different. So, we have $N_{t}=N_{t}^{A}=N_{t}^{B}$, and they can be calculated as

(2) $$N_{t}=N_{0}+\sum_{i=1}^{t}\Delta N\left(i\right)=\left(s-1\right){\left(s+1\right)}^{t}+1.$$

For the number of new edges created at time step *t* in the network $G_{s,r}^{A}\left(t\right)$, we obtain $\Delta E^{A}\left(t\right)=s^{2}\cdot R\left(t-1\right)=s^{2}{\left(s+1\right)}^{t-1}$. According to the iterative construction of $G_{s,r}^{A}\left(t\right)$, we calculate the total number of edges in the network $G_{s,r}^{A}\left(t\right)$ as follows,

(3) $$E_{t}^{A}=E^{A}\left(0\right)+\sum_{i=1}^{t}\Delta E^{A}\left(i\right)=s{\left(s+1\right)}^{t}.$$

Similarly, we examine the number of new edges created at time step *t* in $G_{s,r}^{B}\left(t\right)$, it is equal to $\Delta E^{B}\left(t\right)=\left[s^{2}\left(s-1\right)/2\right]\cdot R\left(t-1\right)=\left[s^{2}\left(s-1\right)/2\right]\cdot {\left(s+1\right)}^{t-1}$, thus, the total number of edges in $G_{s,r}^{B}\left(t\right)$ is equal to

(4) $$E_{t}^{B}=E^{B}\left(0\right)+\sum_{i=1}^{t}\Delta E^{B}\left(i\right)=\frac{s(s-1)}{2}{\left(s+1\right)}^{t}.$$

Therefore, we can obtain the range of the number of edges of the generalized weighted Koch network $G_{s,r}\left(t\right)$, which satisfies $s{\left(s+1\right)}^{t}\leq E_{t}\leq \frac{s(s-1)}{2}{\left(s+1\right)}^{t}$.

Referring to Ref. [22], the average degree of $G_{s,r}^{A}\left(t\right)$ is ${\langle k\rangle }_{A}\to 2s/\left(s-1\right)$ for t→∞. On the other hand, the solution of average degree of $G_{s,r}^{B}\left(t\right)$ is

(5) $${\langle k\rangle }_{B}=\frac{2E_{t}^{B}}{N_{t}^{B}}=\frac{s\left(s-1\right){\left(s+1\right)}^{t}}{\left(s-1\right){\left(s+1\right)}^{t}+1},$$

where ${\langle k\rangle }_{B}$ is approximately *s* for a large *t*, which shows that the two networks $G_{s,r}^{A}\left(t\right)$ and $G_{s,r}^{B}\left(t\right)$ are sparse networks according to the standard proposed in the literature [24] because the condition $E_{t}\ll N_{t}\left(N_{t}-1\right)/2$ is clearly established. Therefore, we obtain that the range of average degree of the generalized weighted Koch network $G_{s,r}\left(t\right)$ satisfies 2s/(s−1)≤〈k〉≤s.

## 3. Topological Properties and ATT

Next, we discuss some relevant topological characteristics of our network, including degree distribution, clustering coefficient, diameter and average weighted shortest path, and give the lower bound of the ATT for our networks.

### 3.1. Degree Distribution

The degree distribution P(k) is a physical quantity that describes the overall characteristics of the network. It is defined as the probability that a randomly selected node in the graph has exactly *k* associated edges. Cumulative degree distribution $P_{cum}\left(k\right)$ is defined as the probability that the degree of a node is greater than or equal to *k*, that is, $P_{cum}\left(k\right)=\sum_{k^{\prime }=k}^{\infty }P\left(k^{\prime }\right)$. Many networks are regarded as scale-free when their cumulative degree distribution approximately follows a power–law distribution $P_{cum}\left(k\right)∼k^{1-\gamma }$ and the power exponent γ lies between 2 and 3 [25].

**Theorem** **1.** *The cumulative degree distribution of the network $G_{s,r}^{B}\left(t\right)$ obeys a power law distribution*

(6) $$\begin{array}{c} P_{cum}^{B}\left(k\right)={\left(s-1\right)}^{\frac{\ln(s+1)}{\ln2}}k^{1-\gamma },\gamma =1+\frac{\ln(s+1)}{\ln2}, \end{array}$$

*and $G_{s,r}^{B}\left(t\right)$ is a scale-free network if, and only if, s=3.*

**Proof.**  Let $k_{i}^{B}\left(t\right)$ be the degree of node *i* in network $G_{s,r}^{B}\left(t\right)$ at time step *t*. When node *i* joins the network at time step $t_{i}$, there is only one $K_{s}$ connected to it, so we have $k_{i}^{B}\left(t_{i}\right)=s-1$. We can find that the degree of node *i* depends on the number of cluster $K_{s}$, including it. Let $R^{B}\left(i,t\right)$ be the number of cluster $K_{s}$ including node *i* at time step *t*. We can establish the following relation $R^{B}\left(i,t\right)=2R^{B}\left(i,t-1\right)$, considering the initial condition $R^{B}\left(i,t_{i}\right)=1$, so $R^{B}\left(i,t\right)=2^{t-t_{i}}$. Further, there is a relationship between $k_{i}^{B}\left(t\right)$ and $R^{B}\left(i,t\right)$,

(7) $$k_{i}^{B}\left(t\right)=\left(s-1\right)R^{B}\left(i,t\right)=\left(s-1\right)2^{t-t_{i}}.$$

We know that the degree of node *i* satisfies $k_{i}^{B}\left(t\right)=2k_{i}^{B}\left(t-1\right)$, which shows that the degree spectrum of $G_{s,r}^{B}\left(t\right)$ is discrete. Table 1 lists the degree spectrum of the two networks $G_{s,r}^{A}\left(t\right)$ and $G_{s,r}^{B}\left(t\right)$, and $n^{Z}\left(k_{i}\right)\left(Z=A,B\right)$ represents the number of nodes with degree $k_{i}$.

Analyzing the degree spectrum of $G_{s,r}^{B}\left(t\right)$, we can obtain its cumulative degree distribution,

(8) $$P_{cum}^{B}\left(k\right)=\frac{1}{N_{t}^{B}}\sum_{k^{\prime }=k}^{\infty }n^{B}\left(k_{i}^{{}^{\prime }B}\right)=\frac{\left(s-1\right){\left(s+1\right)}^{t_{i}}+1}{\left(s-1\right){\left(s+1\right)}^{t}+1},$$

we can solve for $t_{i}=t-\frac{\ln k/(s-1)}{\ln2}$ from Equation (7) and substitute $t_{i}$ into Equation (8), then

(9) $$\begin{array}{cc} P_{cum}^{B}\left(k\right) & =\frac{\left(s-1\right){\left(s+1\right)}^{t}{\left[k/\left(s-1\right)\right]}^{-\frac{\ln(s+1)}{\ln2}}+1}{\left(s-1\right){\left(s+1\right)}^{t}+1}\\ \; & \approx {\left(s-1\right)}^{\frac{\ln(s+1)}{\ln2}}k^{-\frac{\ln(s+1)}{\ln2}}, \end{array}$$

when *t* is large enough, the cumulative degree distribution follows a power law with exponent γ=1+ln(s+1)/ln2∈[2,3] if, and only if, 1≤s≤3, considering that the range of the parameter *s* is s≥3, so the network $G_{s,r}^{B}\left(t\right)$ is a scale-free network when s=3. On the other hand, the cumulative degree distribution of $G_{s,r}^{A}\left(t\right)$ can be found in [22]. It is expressed as $P_{cum}^{A}\left(k\right)=2^{\frac{\ln(s+1)}{\ln2}}k^{-\frac{\ln(s+1)}{\ln2}}$, which shows that the network $G_{s,r}^{A}\left(t\right)$ also satisfies the properties of the scale-free network when s=3. Therefore, we can judge that the generalized weighted Koch network $G_{s,r}\left(t\right)$ has a remarkable scale-free property when s=3. When s>3, the degree distribution of $G_{s,r}\left(t\right)$ obeys a power-law distribution $k^{1-\gamma }$ and the exponent is $\gamma =1+\frac{\ln(s+1)}{\ln2}$. □

### 3.2. Clustering Coefficient

The overall clustering coefficient of a network is used to quantity the ability of the network to agglomerate, while the local clustering coefficient can measure the agglomeration near each node in the network. The local clustering coefficient $c_{i}$ corresponding to node *i* is defined as the ratio between the number of existing edges $e_{i}$ connecting its $k_{i}$ neighbors and the number of all possible edges $k_{i}\left(k_{i}-1\right)/2$ between them, that is, $c_{i}=2e_{i}/k_{i}\left(k_{i}-1\right)$ [26]. The overall clustering coefficient of the network is denoted as $C=\frac{1}{|N_{t}|}\sum_{i\in V(G)}c_{i}$; it is defined as the average of $c_{i}$ over all nodes in the network.

**Theorem** **2.** *For the network $G_{s,r}^{B}\left(t\right)$, the solution of the overall clustering coefficient of the whole network satisfies*

(10) $$\begin{array}{cc} C^{B} & \to \frac{2s+1}{2(s+1)},\;\;for\;\;t\to \infty . \end{array}$$

**Proof.**  According to the structure of the network $G_{s,r}^{B}\left(t\right)$, it can be judged that the nodes with the same degree have the same local clustering coefficient. Therefore, Table 2 shows the local clustering coefficient and the corresponding number of nodes. $n^{B}\left(k_{i}^{B}\right)$ represents the number of nodes with degree $k_{i}^{B}$, and $c(k_{i}^{B})$ is the local clustering coefficient of each node with degree $k_{i}^{B}$. Therefore, the overall clustering coefficient $C^{B}$ of $G_{s,r}^{B}\left(t\right)$ is

(11) $$\begin{array}{cc} C^{B} & =\frac{1}{N_{t}^{B}}\sum_{k_{i}^{B}}c\left(k_{i}^{B}\right)\cdot n^{B}\left(k_{i}^{B}\right)\\ \; & =\frac{1}{\left(s-1\right){\left(s+1\right)}^{t}+1}{[s\left(s-1\right)\left(s+1\right)}^{t-1}+\frac{s\left(s-2\right)\left(s-1\right){\left(s+1\right)}^{t-2}}{2(s-1)-1}\\ \; & \;\;+\frac{s\left(s-2\right)\left(s-1\right){\left(s+1\right)}^{t-3}}{2^{2}\left(s-1\right)-1}+\cdots +\frac{s(s-2)}{2^{t}\left(s-1\right)-1}]\\ \; & \to \frac{2s+1}{2(s+1)}. \end{array}$$

In the limit of t→∞, we have $C^{B}\to 1$, which indicates that the network $G_{s,r}^{B}\left(t\right)$ is high clustered. We can control the clustering coefficient by adjusting parameter *s*. For network $G_{s,r}^{A}\left(t\right)$, when s=3, the clustering coefficient $C^{A}$ is approximately $k^{-1}$ in the large limit of *t*; otherwise, the clustering coefficient of $G_{s,r}^{A}\left(t\right)$ in other cases is always equal to zero. Therefore, the generalized weighted Koch networks transition from low clustered to high clustered, as the probability *p* keeps increasing in the interval [0,1]. □

### 3.3. Diameter

The diameter $D_{max}\left(t\right)$ of a network is used to measure information transmission delays and find vital nodes in the network. It is defined as the largest distance between any pair of nodes. Let $D_{max}^{A}\left(t\right)$ and $D_{max}^{B}\left(t\right)$ denote the diameters of $G_{s,r}^{A}\left(t\right)$ and $G_{s,r}^{B}\left(t\right)$, respectively.

**Theorem** **3.** *The diameter of the network $G_{s,r}^{A}\left(t\right)$ is equal to*

(12) $$D_{max}^{A}\left(t\right)=\left\{\begin{array}{cc} (\frac{1}{2}+t)s, & i\;\;is\;even;\\ \left(\frac{1}{2}+t\right)\left(s-1\right), & i\;\;is\;odd. \end{array}\right.$$

*The diameter of the network $G_{s,r}^{B}\left(t\right)$ is*

(13) $$D_{max}^{B}\left(t\right)=2t+1.$$

**Proof.**  By the topological structure of $G_{s,r}^{A}\left(t\right)$, we find that a node on the cycle $C_{s}$ can only reach another node through a path on the cycle, so we discuss the diameter of the network $G_{s,r}^{A}\left(t\right)$ according to the parity of the number of nodes.

Case 1. When *s* is even, we assume that the two nodes with the farthest distance in the network $G_{s,r}^{A}\left(t-1\right)$ are $x_{1}$ and $x_{2}$. In network $G_{s,r}^{A}\left(t\right)$, $y_{1}$ is the farthest node to $x_{1}$ among the new neighbors of $x_{1}$, and $y_{2}$ is the farthest node to $x_{2}$ among the new neighbors of $x_{2}$. New neighbors refer to those nodes generated at time step *t* among all neighbors. Then, the diameter of $G_{s,r}^{A}\left(t\right)$ refers to the distance between nodes $y_{1}$ and $y_{2}$; we can obtain the following equation,

(14) $$D_{max}^{A}\left(t\right)=D_{max}^{A}\left(t-1\right)+s,$$

with the help of $D_{max}^{A}\left(0\right)=s/2$, we can obtain

(15) $$D_{max}^{A}\left(t\right)=\left(\frac{1}{2}+t\right)s.$$

Case 2. When *s* is odd, the diameter of the network $G_{s,r}^{A}\left(t\right)$ at two consecutive time steps t−1 and *t* satisfies the following relationship:

(16) $$D_{max}^{A}\left(t\right)=D_{max}^{A}\left(t-1\right)+s-1,$$

where it is obvious that the diameter of the smallest network $G_{s,r}^{A}\left(0\right)$ is $D_{max}^{A}\left(0\right)=\left(s-1\right)/2$, so the expression of diameter can be obtained according to the above recursive formula,

(17) $$D_{max}^{A}\left(t\right)=\left(\frac{1}{2}+t\right)\left(s-1\right).$$

Considering the construction algorithm of $G_{s,r}^{B}\left(t\right)$, the diameters of $G_{s,r}^{B}\left(t\right)$ and $G_{s,r}^{B}\left(t-1\right)$ at two consecutive time steps have the following rule:

(18) $$D_{max}^{B}\left(t\right)=D_{max}^{B}\left(t-1\right)+2,$$

the initial condition is $D_{max}^{B}\left(0\right)=1$, then for any t≥0, we have

(19) $$D_{max}^{B}\left(t\right)=2t+1.$$

Theorem 3 shows that the propagation efficiency of $G_{s,r}^{B}\left(t\right)$ is more efficient than that of $G_{s,r}^{A}\left(t\right)$. Among all generalized weighted Koch networks, the network $G_{s,r}^{A}\left(t\right)$ has the largest diameter and the network $G_{s,r}^{B}\left(t\right)$ has the smallest diameter. Thus, we can determine that the diameter of the generalized weighted Koch network satisfies $2t+1\leq D_{max}\left(t\right)\leq \left(\frac{1}{2}+t\right)s$. □

### 3.4. Average Weighted Shortest Path

In this subsection, we take the weight into account to examine the shortest path between two nodes in our networks. The average weighted shortest path is defined as $L_{t}=\frac{2}{N_{t}\left(N_{t}-1\right)}D_{tot}\left(t\right)$, where $D_{tot}\left(t\right)=\sum_{i,j\in G_{s,r}\left(t\right),i\neq j}d_{ij}\left(t\right)$, $d_{ij}\left(t\right)$ denotes the weighted shortest path connecting node *i* and *j* in network $G_{s,r}\left(t\right)$ [27,28]. We only determine the lower bound of $L_{t}$ for our network $G_{s,r}\left(t\right)$ by calculating the average weighted shortest path of $G_{s,r}^{B}\left(t\right)$.

**Theorem** **4.** *For the networks $G_{s,r}^{B}\left(t\right)$, when r=1, its average weighted shortest path is*

(20) $$L_{t}^{B}=\frac{2[A_{1}{\left(s+1\right)}^{t}+A_{2}{\left(s+1\right)}^{2t}+A_{3}t{\left(s+1\right)}^{2t}]}{\left[\left(s-1\right){\left(s+1\right)}^{t}+1\right]\left(s-1\right){\left(s+1\right)}^{t}},$$

*when r≠1, then*

(21) $$L_{t}^{B}=\frac{2[A_{4}{\left(sr+1\right)}^{t}+A_{5}{\left(s+1\right)}^{2t}+A_{6}{\left(sr+1\right)}^{t}{\left(s+1\right)}^{t}]}{\left[\left(s-1\right){\left(s+1\right)}^{t}+1\right]\left(s-1\right){\left(s+1\right)}^{t}},$$

*the values $A_{1}-A_{6}$ are in Appendix A. They are constants that depend on the parameter s and the weight r, and do not depend on the time step t.*

**Proof.**  The recursive construction of the network allows us to calculate the $D_{tot}\left(t\right)$. The network $G_{s,r}^{B}\left(t+1\right)$ can be divided into s+1 branches, which we label as $G_{s,r}^{B,n}\left(t\right)$ for n=1,2,⋯,s,s+1, the center branch $G_{s,r}^{B,1}\left(t\right)$ is a copy of $G_{s,r}^{B}\left(t\right)$, and $G_{s,r}^{B,2}\left(t\right)$, $G_{s,r}^{B,3}\left(t\right)$, ⋯, $G_{s,r}^{B,s+1}\left(t\right)$ have the same structure as $G_{s,r}^{B}\left(t\right)$, but their edge weights are scaled by a factor of *r*. We denote the connected nodes as $W_{1},W_{2},\cdots \;,W_{s}$, which connect the copy $G_{s,r}^{B,1}\left(t\right)$ and other copies $G_{s,r}^{B,n^{\prime }}\left(t\right)$, $n^{\prime }=2,3,\cdots \;,s+1$. Therefore, the total of the shortest distances $D_{tot}\left(t+1\right)$ satisfies the following relation,

(22) $$D_{tot}\left(t+1\right)=\left(sr+1\right)D_{tot}\left(t\right)+\Omega_{t},$$

where $\Omega_{t}$ is the sum over all shortest paths whose nodes are not in the same copy of $G_{s,r}^{B}\left(t\right)$, that is to say, the paths in $\Omega_{t}$ must all go though at least one of the *s* connected nodes $W_{1}$, $W_{2}$, ⋯, $W_{s}$. The first term on Equation (22) is the sum of weighted shortest path linking node *i* and *j* in every $G_{s,r}^{B,n^{\prime }}\left(t\right)$, $n^{\prime }=2,3,\cdots ,s+1$. Considering the scaling of the edges, we have

(23) $$\begin{array}{cc} \sum_{n=1}^{s+1}\sum_{i,j\in G_{s,r}^{B,n}\left(t\right)}d_{ij} & =\sum_{i,j\in G_{s,r}^{B,1}\left(t\right)}d_{ij}+\sum_{n^{\prime }=2}^{s+1}\sum_{i,j\in G_{s,r}^{B,n^{\prime }}\left(t\right)}d_{ij}\\ \; & =\sum_{i,j\in G_{s,r}^{B}\left(t\right)}d_{ij}+rs\sum_{i,j\in G_{s,r}^{B}\left(t\right)}d_{ij}\\ \; & =\left(sr+1\right)D_{tot}\left(t\right). \end{array}$$

Next, the analytical expression for $\Omega_{t}$ is not difficult to find. We denote $\Omega_{t}^{\alpha \beta }$ as the sum of all shortest paths with nodes in $G_{s,r}^{B,\alpha }\left(t\right)$ and $G_{s,r}^{B,\beta }\left(t\right)$; there are two different situations that need to be discussed. Let $\Omega_{t}^{\alpha \beta }$ denote the sum of the distances of the nodes in $G_{s,r}^{B,n^{\prime }}$ to the nodes in $G_{s,r}^{B,1}$ for $n^{\prime }=2,3,\cdots ,s+1$. Moreover, $\Omega_{t}^{\alpha \gamma }$ represents the sum of nodes in different $G_{s,r}^{B,n^{\prime }}$, which must pass through $G_{s,r}^{B,1}$, but their end node is not in $G_{s,r}^{B,1}$. Thus, we have

(24) $$\Omega_{t}=s\Omega_{t}^{\alpha \beta }+\frac{s(s-1)}{2}\Omega_{t}^{\alpha \gamma }.$$

It can be seen from the above formula that we need to calculate $\Omega_{t}^{\alpha \beta }$ and $\Omega_{t}^{\alpha \gamma }$ to obtain $\Omega_{t}$. We define a variable

(25) $$\Delta_{t}=\sum_{i\in G_{s,r}^{B}\left(t\right),i\neq W_{2}}d_{iW_{2}},$$

which represents the sum of the distances from all nodes in $G_{s,r}^{B}\left(t\right)$ to node $W_{2}$; specifically, we can obtain the following result for $G_{s,r}^{B}\left(1\right)$,

(26) $$\Delta_{1}=\left(r+1\right){\left(s-1\right)}^{2}+\left(s-1\right)r+\left(s-1\right).$$

Considering the self-similar structure at time step *t*, we know that the quantity $\Delta_{t}$ evolves recursively as

(27) $$\begin{array}{cc} \Delta_{t} & =r\Delta_{t-1}+\left(s-1\right)\left(r\Delta_{t-1}+N_{t-1}-1\right)+\Delta_{t-1}\\ \; & =\left(sr+1\right)\Delta_{t-1}+{\left(s-1\right)}^{2}{\left(s+1\right)}^{t-1}. \end{array}$$

We obtained the recursive relation of $\Delta_{t}$, and combined with the initial condition $\Delta_{1}$ given by Equation (26), we can calculate the expression of $\Delta_{t}$,

(28) $$\Delta_{t}=\left\{\begin{array}{cc} \left[{\left(s-1\right)}^{2}t+s^{2}-1\right]{\left(s+1\right)}^{t-1}, & r=1;\\ {\left(sr+1\right)}^{t}\frac{s-1-(s^{2}-s)r}{s(1-r)}+\frac{{\left(s-1\right)}^{2}}{s(1-r)}{\left(s+1\right)}^{t}, & r\neq 1. \end{array}\right.$$

Then, we calculate the two variables $\Omega_{t}^{\alpha \beta }$ and $\Omega_{t}^{\alpha \gamma }$ through the variable $\Delta_{t}$, and the following two cases are discussed.

Case 1. This situation shows that one of α and β must be equal to 1 because only copy $G_{s,r}^{B,1}\left(t\right)$ and other copies $G_{s,r}^{B,n^{\prime }}\left(t\right)$ have a common node. Let $G_{s,r}^{B,\alpha }\left(t\right)$ and $G_{s,r}^{B,\beta }\left(t\right)$ have a common node $W_{k}$, where α,β∈[1,s+1],α≠β. For two nodes $i\in G_{s,r}^{B,\alpha }\left(t\right)$, $j\in G_{s,r}^{B,\beta }\left(t\right)$ and $W_{k}\neq i,j$, we have

(29) $$\begin{array}{cc} \Omega_{t}^{\alpha \beta } & =\sum_{\begin{array}{c} i\in G_{s,r}^{\alpha }\left(t\right),j\in G_{s,r}^{\beta }\left(t\right)\\ i,j\neq W_{2} \end{array}}d_{ij}\\ \; & =\sum_{\begin{array}{c} i\in G_{s,r}^{\alpha }\left(t\right),j\in G_{s,r}^{\beta }\left(t\right)\\ i,j\neq W_{2} \end{array}}\left(d_{iW_{2}}+d_{jW_{2}}\right)\\ \; & =\left(N_{t}-1\right)\sum_{\begin{array}{c} i\in G_{s,r}^{\alpha }\left(t\right)\\ i\neq W_{2} \end{array}}d_{iW_{2}}+\left(N_{t}-1\right)\sum_{\begin{array}{c} j\in G_{s,r}^{\beta }\left(t\right)\\ j\neq W_{2} \end{array}}d_{jW_{2}}\\ \; & =\left(r+1\right)\left(N_{t}-1\right)\Delta_{t}. \end{array}$$

Case 2. $G_{s,r}^{B,\alpha }\left(t\right)$ and $G_{s,r}^{B,\gamma }\left(t\right)$ have no common node, so it must cross two nodes $W_{k}$ and $W_{m}$ in copy $G_{s,r}^{B,1}\left(t\right)$ from node *i* to *j*.

(30) $$\begin{array}{cc} \Omega_{t}^{\alpha \gamma } & =\sum_{\begin{array}{c} i\in G_{s,r}^{\alpha }\left(t\right),j\in G_{s,r}^{\gamma }\left(t\right)\\ i,j\neq W_{2} \end{array}}d_{ij}\\ \; & =\sum_{\begin{array}{c} i\in G_{s,r}^{\alpha }\left(t\right),j\in G_{s,r}^{\gamma }\left(t\right)\\ i,j\neq W_{2},m\neq 2 \end{array}}\left(d_{iW_{2}}+d_{W_{2}W_{m}}+d_{W_{i^{\prime }}j}\right)\\ \; & =r\left(N_{t}-1\right)\Delta_{t}+r\left(N_{t}-1\right)\Delta_{t}+{\left(N_{t}-1\right)}^{2}\\ \; & =2r\left(N_{t}-1\right)\Delta_{t}+{\left(N_{t}-1\right)}^{2}. \end{array}$$

where $d_{W_{2}W_{m}}=1$ is used, and $W_{m}$ refers to other nodes on $G_{s,r}^{B}\left(t-1\right)$ except node $W_{2}$. Substituting Equations (29) and (30) into Equation (24), we have

(31) $$\Omega_{t}=s\left[r\left(N_{t}-1\right)\Delta_{t}+\left(N_{t}-1\right)\Delta_{t}\right]+\frac{s(s-1)}{2}\left[2r\left(N_{t}-1\right)\Delta_{t}+{\left(N_{t}-1\right)}^{2}\right],$$

Considering Equations (2) and (28), then substituting them into Equation (31), we obtain the following.

If r=1, then

(32) $$\begin{array}{cc} \Omega_{t} & =\left[s{\left(s-1\right)}^{3}t+\frac{\left(3s^{2}+s\right){\left(s-1\right)}^{2}}{2}\right]{\left(s+1\right)}^{2t}. \end{array}$$

If r≠1, we have

(33) $$\begin{array}{cc} \Omega_{t} & =\frac{{\left(s-1\right)}^{2}\left(1-s^{2}r^{2}\right)}{1-r}{\left(sr+1\right)}^{t}{\left(s+1\right)}^{t}+\frac{\left(s^{2}-s\right)r+s^{2}+s-2}{2(1-r)}{\left(s-1\right)}^{2}{\left(s+1\right)}^{2t}. \end{array}$$

According to Equation (22) and the initial condition $D_{tot}\left(0\right)=\frac{s(s-1)}{2}$, the result of $D_{tot}\left(t\right)$ can be obtained by the recursive formula. If r=1, we have

(34) $$\begin{array}{cc} D_{tot}\left(t\right) & ={\left(s+1\right)}^{t}\frac{s^{2}\left(s^{2}-1\right)+2{\left(s-1\right)}^{3}\left(s+1\right)-\left(3s^{2}+s\right){\left(s-1\right)}^{2}}{2s(s+1)}\\ \; & \;\;\;+{\left(s+1\right)}^{2t}\frac{-2{\left(s-1\right)}^{3}-2s{\left(s-1\right)}^{3}+\left(3s^{2}+s\right){\left(s-1\right)}^{2}}{2s(s+1)}\\ \; & \;\;\;+t{\left(s+1\right)}^{2t}\frac{{\left(s-1\right)}^{3}}{s+1}. \end{array}$$

When r≠1, we obtain

(35) $$\begin{array}{cc} D_{tot}\left(t\right) & ={\left(sr+1\right)}^{t}\{\frac{s(s-1)}{2}-\frac{{\left(s-1\right)}^{2}\left(1-s^{2}r^{2}\right)}{s(1-r)(sr+1)}\\ \; & \;\;\;+\frac{\left[\left(s^{2}-s\right)r+s^{2}+s-2\right]{\left(s-1\right)}^{2}{\left(s+1\right)}^{2}}{2\left(1-r\right)[sr+1-{\left(s+1\right)}^{2}]\left(sr+1\right)}\}\\ \; & \;\;\;+{\left(s+1\right)}^{2t}\frac{\left[\left(s^{2}-s\right)r+s^{2}+s-2\right]{\left(s-1\right)}^{2}}{2\left(1-r\right)[sr+1-{\left(s+1\right)}^{2}]}\\ \; & \;\;\;+{\left(sr+1\right)}^{t}{\left(s+1\right)}^{t}\frac{{\left(s-1\right)}^{2}\left(1-s^{2}r^{2}\right)}{s(1-r)(sr+1)}. \end{array}$$

Further, according to the equation $L_{t}=\frac{2}{N_{t}\left(N_{t}-1\right)}D_{tot}\left(t\right)$, substituting Equations (34) and (35) into it, we can obtain the average weighted shortest path for r=1,

(36) $$\begin{array}{c} L_{t}^{B}=\frac{2[A_{1}{\left(s+1\right)}^{t}+A_{2}{\left(s+1\right)}^{2t}+A_{3}t{\left(s+1\right)}^{2t}]}{\left[\left(s-1\right){\left(s+1\right)}^{t}+1\right]\left(s-1\right){\left(s+1\right)}^{t}}. \end{array}$$

For r≠1, we can obtain

(37) $$\begin{array}{c} L_{t}^{B}=\frac{2[A_{4}{\left(sr+1\right)}^{t}+A_{5}{\left(s+1\right)}^{2t}+A_{6}{\left(sr+1\right)}^{t}{\left(s+1\right)}^{t}]}{\left[\left(s-1\right){\left(s+1\right)}^{t}+1\right]\left(s-1\right){\left(s+1\right)}^{t}}, \end{array}$$

where $A_{1},A_{2},A_{3},A_{4},A_{5},A_{6}$ are constants that do not depend on the time step *t*, they are only related to *s* and *r*. Appendix A contains the detailed values of $A_{1}-A_{6}$. Therefore, we can determine that the lower bound of the average weighted shortest path of network $G_{s,r}\left(t\right)$ is equal to $L_{t}^{B}$. □

### 3.5. ATT on Random Walk with Weight

Next, we derive analytically the average trapping time on random walk with weight and show how it scales with the network order and parameters *r* and *s*. The strength of a node integrates the information concerning its connectivity and the weights of its edges. Let $s_{i}=\sum_{j\in N(i)}w_{ij}$ be the strength of node *i*. The walker starting from a given node *i* moves to its neighbor node *j* with probability $p_{i\to j}$ at each step, and the transition probability from node *i* to *j* is

(38) $$p_{i\to j}=\frac{w_{ij}}{s_{i}}=\frac{w_{ij}}{\sum_{j\in N(i)}w_{ij}},$$

where N(i) is the neighbors of node *i*. For the convenience of description, let us denote all nodes in $G_{s,r}\left(t-1\right)$ by $1,2,\cdots ,N_{t-1}-1,N_{t-1}$, and $N_{t-1}+1,N_{t-1}+2,\cdots ,N_{t}-1,N_{t}$ represent other nodes generated at time step *t*. Let $T_{i}^{\left(t\right)}$ be the MFPT from node *i* to the trap. ${\langle T\rangle }_{t}$ denotes the ATT, which is defined as the mean of $T_{i}^{\left(t\right)}$ starting from all sources of nodes over the whole network to the trap node. It is the core issue considered in this subsection. By definition, ${\langle T\rangle }_{t}$ is given by

(39) $${\langle T\rangle }_{t}=\frac{1}{N_{t}-1}\sum_{i=2}^{N_{t}}T_{i}^{\left(t\right)},$$

and further, we denote the sum of MFPTs for all nodes to absorption at the trap located the one of the nodes of G(0) as $T_{tot}\left(t\right)$, that is

(40) $$T_{tot}\left(t\right)=\sum_{i=2}^{N_{t}}T_{i}^{\left(t\right)}.$$

**Theorem** **5.** *Let r>0 be a weight factor. When r=1, the average trapping time of the network $G_{s,r}^{B}\left(t\right)$ is*

(41) $$\begin{array}{cc} {\langle T\rangle }_{t}^{B} & =\frac{1}{\left(s-1\right){\left(s+1\right)}^{t}}\left[B_{1}{\left(1+s\right)}^{t}+B_{2}{\left(1+s\right)}^{2t}-B_{3}t{\left(1+s\right)}^{t}\right], \end{array}$$

*when r≠1, then*

(42) $$\begin{array}{c} {\langle T\rangle }_{t}^{B}=\frac{1}{\left(s-1\right){\left(s+1\right)}^{t}}\left[B_{4}{\left(1+sr\right)}^{t}+B_{5}{\left(1+sr\right)}^{t}{\left(1+s\right)}^{t}-B_{6}{\left(1+s\right)}^{t}\right], \end{array}$$

*where $B_{1}-B_{6}$ is in Appendix B. The relationship between the average trapping time and the network order $N_{t}$ can be expressed as*

(43) $${\langle T\rangle }_{t}∼\left\{\begin{array}{cc} N_{t}, & r=1;\\ N_{t}^{\log_{s+1}\left(rs+1\right)}, & r\neq 1. \end{array}\right.$$

**Proof.**  For a certain time step $t_{i}$, the nodes generated at time step $t_{i}$ are called new nodes, and the nodes added to the network before time step $t_{i}$ are called old nodes. Then, we let *X* be the MFPT starting from node *i* to any of its $k_{i}\left(t-1\right)$ old neighbors, and let *Y* be the MFPT from any of new neighbors of node *i* to one of its $k_{i}\left(t-1\right)$ old neighbors; thus, we can establish the following relations among *X* and *Y*,

(44) $$\left\{\begin{array}{c} X=\frac{1}{r+1}+\frac{r}{r+1}\left(1+Y\right),\\ Y=\frac{1}{s-1}\left(1+X\right)+\frac{s-2}{s-1}\left(1+Y\right). \end{array}\right.$$

We obtain that the result is X=1+sr. Upon the evolution of the weighted network from time step *t* to time step t+1, the trapping time for an arbitrary node *i* increases by a factor of 1+sr, that is

(45) $$T_{i}^{\left(t+1\right)}=\left(1+sr\right)T_{i}^{\left(t\right)}.$$

**Figure 4:**
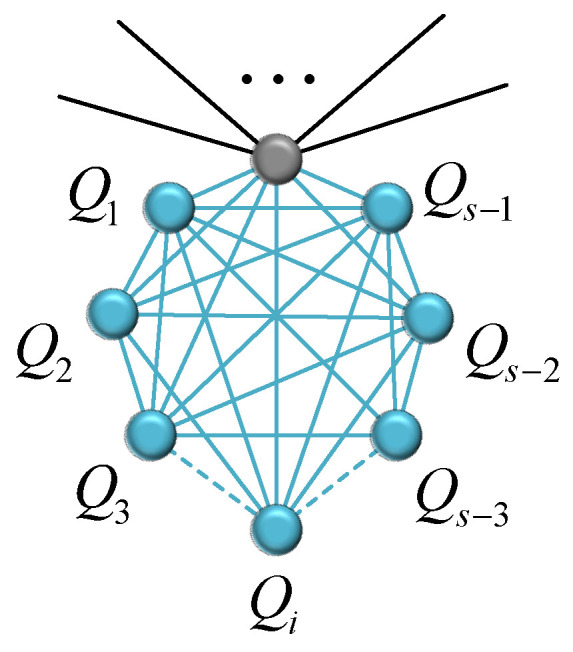
The positional relationship between node *v* and its neighbors.

Next, we consider the MFPT of all nodes in light of the classification of new nodes and old nodes, which is written as the following formula:

(46) $$T_{t,tot}^{\left(t\right)}=T_{t-1,tot}^{\left(t\right)}+{\bar{T}}_{t,tot}^{\left(t\right)}=\left(1+sr\right)T_{t-1,tot}^{\left(t-1\right)}+{\bar{T}}_{t,tot}^{\left(t\right)},$$

where ${\bar{T}}_{t,tot}^{\left(t\right)}$ is the sum of MFPTs for all new nodes. Equation (46) shows that the focus of our calculation is ${\bar{T}}_{t,tot}^{\left(t\right)}$. According to the construction of network $G_{s,r}^{B}\left(t\right)$, as shown in Figure 4, for the new complete graph $K_{s}$ involving a node *v*, the first passage times for its s−1 nodes $Q_{1}$, $Q_{2}$, ⋯, and $Q_{s-1}$, and that of its old node *v* follow the relations,

(47) $$\left\{\begin{array}{c} T\left(Q_{1}\right)=1+\frac{1}{s-1}\left[T\left(v\right)+T\left(Q_{2}\right)+T\left(Q_{3}\right)+\cdots +T\left(Q_{s-1}\right)\right],\\ T\left(Q_{2}\right)=1+\frac{1}{s-1}\left[T\left(v\right)+T\left(Q_{1}\right)+T\left(Q_{3}\right)+\cdots +T\left(Q_{s-1}\right)\right],\\ \cdots \cdots \\ T\left(Q_{s-1}\right)=1+\frac{1}{s-1}\left[T\left(v\right)+T\left(Q_{1}\right)+T\left(Q_{2}\right)+\cdots +T\left(Q_{s-2}\right)\right]. \end{array}\right.$$

According to the above equations, we obtain

(48) $$T\left(Q_{1}\right)+T\left(Q_{2}\right)+\cdots +T\left(Q_{s-1}\right)={\left(s-1\right)}^{2}+\left(s-1\right)T\left(v\right),$$

summing Equation (48) over all the $R\left(t\right)={\left(s+1\right)}^{t}$ old $K_{s}$ pre-existing at the time step *t*. Let V(t) be the set of all nodes of the $G_{s,r}^{B}\left(t\right)$. It contains all old nodes in network $G_{s,r}^{B}\left(t\right)$,

(49) $$\begin{array}{cc} {\bar{T}}_{t+1,tot}^{\left(t+1\right)} & =s{\left(s-1\right)}^{2}R\left(t\right)+\sum_{i\in V(t)}\left[\left(s-1\right)\cdot R^{B}\left(i,t\right)\cdot T_{i}^{\left(t+1\right)}\right]\\ \; & =s{\left(s-1\right)}^{2}{\left(s+1\right)}^{t}+\left(s-1\right){\bar{T}}_{t,tot}^{\left(t+1\right)}+2\left(s-1\right){\bar{T}}_{t-1,tot}^{\left(t+1\right)}\\ \; & \;\;\;+\cdots +2^{t}\left(s-1\right){\bar{T}}_{0,tot}^{\left(t+1\right)}. \end{array}$$

Similarly, it is not difficult to write ${\bar{T}}_{t,tot}^{\left(t\right)}$ as

(50) $$\begin{array}{cc} {\bar{T}}_{t,tot}^{\left(t\right)} & =s{\left(s-1\right)}^{2}{\left(s+1\right)}^{t-1}+\left(s-1\right){\bar{T}}_{t-1,tot}^{\left(t\right)}+2\left(s-1\right){\bar{T}}_{t-2,tot}^{\left(t\right)}\\ \; & \;\;\;+\cdots +2^{t-1}\left(s-1\right){\bar{T}}_{0,tot}^{\left(t\right)}. \end{array}$$

Multiplying Equation (50) with 2(1+sr) and subtracting the result from Equation (49), we obtain

(51) $${\bar{T}}_{t+1,tot}^{\left(t+1\right)}=\left(1+sr\right)\left(1+s\right){\bar{T}}_{t,tot}^{\left(t\right)}+s{\left(s-1\right)}^{2}\left(s-2sr-1\right){\left(s+1\right)}^{t-1}.$$

Considering ${\bar{T}}_{1,tot}^{\left(1\right)}=\frac{4s^{2}-32s+16}{s-4}$, substituting ${\bar{T}}_{1,tot}^{\left(1\right)}$ into Equation (51), we can compute Equation (51) to yield

(52) $$\begin{array}{cc} {\bar{T}}_{t,tot}^{\left(t\right)} & =\left[\frac{4s^{2}-32s+16}{s-4}+\frac{s{\left(s-1\right)}^{2}\left(s-2sr-1\right)}{(1+sr)(1+s)-s-1}\right]{\left(1+sr\right)}^{t-1}{\left(1+s\right)}^{t-1}\\ \; & \;\;\;-\frac{s{\left(s-1\right)}^{2}\left(s-2sr-1\right)}{(1+sr)(1+s)-s-1}{\left(s+1\right)}^{t-1}, \end{array}$$

substituting Equation (52) into Equation (46) and taking the initial value $T_{1,tot}^{\left(1\right)}=\frac{4s^{2}-40s+24}{s-4}$ into consideration. When r=1, we have

(53) $$\begin{array}{cc} T_{t,tot}^{\left(t\right)} & ={\left(1+s\right)}^{t}\left[\frac{4s^{2}-40s+24}{\left(s-4)(s+1\right)}-\frac{{\left(s-1\right)}^{2}}{s(1+s)}-\frac{4s^{2}-32s+16}{s^{2}-4s}\right]\\ \; & \;\;\;+{\left(1+s\right)}^{2t}\left[\frac{4s^{2}-32s+16}{\left(s^{2}-4s\right)\left(1+s\right)}-\frac{{\left(s-1\right)}^{2}}{s(1+s)}\right]\\ \; & \;\;\;+t{\left(1+s\right)}^{t}\frac{{\left(s-1\right)}^{2}}{1+s}. \end{array}$$

If r≠1, we obtain

(54) $$\begin{array}{cc} T_{t,tot}^{\left(t\right)} & ={\left(1+sr\right)}^{t-1}\frac{4s^{2}-40s+24}{s-4}+[\frac{4s^{2}-32s+16}{s-4}\\ \; & \;\;\;+\frac{s{\left(s-1\right)}^{2}\left(s-2sr-1\right)}{(1+sr)(1+s)-s-1}{]\left(1+sr\right)}^{t-1}\cdot \frac{\left(1+s\right)[{\left(1+s\right)}^{t-1}-1]}{s}\\ \; & \;\;\;-\frac{s{\left(s-1\right)}^{2}\left(s-2st-1\right)}{(1+sr)(1+s)-s-1}\cdot \frac{{\left(1+sr\right)}^{t-1}\left(1+s\right)-{\left(1+s\right)}^{t}}{sr-s}. \end{array}$$

Substituting Equations (53) and (54) into Equation (40), if r=1, we obtain

(55) $$\begin{array}{cc} {\langle T\rangle }_{t}^{B} & =\frac{1}{\left(s-1\right){\left(s+1\right)}^{t}}\left[B_{1}{\left(1+s\right)}^{t}+B_{2}{\left(1+s\right)}^{2t}-B_{3}t{\left(1+s\right)}^{t}\right], \end{array}$$

where $B_{1},B_{2},B_{3}$ are constants independent of *t*, so ${\langle T\rangle }_{t}^{B}∼N_{t}$ for t→∞. Additionally, if r≠1, we can obtain

(56) $$\begin{array}{cc} {\langle T\rangle }_{t}^{B} & =\frac{1}{\left(s-1\right){\left(s+1\right)}^{t}}\left[B_{4}{\left(1+sr\right)}^{t}+B_{5}{\left(1+sr\right)}^{t}{\left(1+s\right)}^{t}-B_{6}{\left(1+s\right)}^{t}\right], \end{array}$$

where $B_{4},B_{5},B_{6}$ are parameters that have nothing to do with *t*, as they are only related to parameters *r* and *s*. The detailed values of $B_{1}-B_{6}$ are given in Appendix B. Next, we show how to express ${\langle T\rangle }_{t}^{B}$ in terms of network order $N_{t}$. We can obtain $t=\log_{s+1}\left(\frac{1}{s-1}N_{t}-\frac{1}{s-1}\right)$ from $N_{t}=\left(s-1\right){\left(s+1\right)}^{t}+1$, so we have

(57) $${\langle T\rangle }_{t}^{B}=N_{t}^{\log_{s+1}\left(1+sr\right)},$$

for t→∞. The above results show that ${\langle T\rangle }_{t}^{B}$ grows linearly with the network order when r=1, while ${\langle T\rangle }_{t}^{B}$ grows sub-linearly and super-linearly with $N_{t}$ if r<1 and r>1, respectively. We find that the weight *r* can modify not only the prefactor of ${\langle T\rangle }_{t}^{B}$, but also the scaling of ${\langle T\rangle }_{t}^{B}$. This means that the lower bound of the ATT with the weight of the generalized weighted Koch network $G_{s,r}\left(t\right)$ is ${\langle T\rangle }_{t}^{B}$. □

## 4. Conclusions

In this paper, we construct a class of random networks according to probability *p* and normal Koch networks, and give the necessary and sufficient conditions for such networks to be scale free. For two deterministic network models $G_{s,r}^{A}\left(t\right)$ and $G_{s,r}^{B}\left(t\right)$ corresponding to two extreme conditions p=0 and p=1, we give exact solutions for topological parameters, including the clustering coefficient, diameter, average weighted shortest path and average trapping time. In view of this, we determine the range of topological parameters of random network $G_{s,r}\left(t\right)$. Furthermore, we reveal the effect of weights on ATT, that is, ATT grows linearly with the network order when the weight r=1; otherwise, ATT grows superlinearly and sub-linearly with the network order when r>1 and r<1. These topological features and dynamic characteristics allow us to better understand some fundamental properties of complex networks.

## Figures and Tables

**Table 1:** The degree spectrum of $G_{s,r}^{A}\left(t\right)$ and $G_{s,r}^{B}\left(t\right)$.

*t*	$k_{i}^{A}$	$n^{A}\left(k_{i}^{A}\right)$	$k_{i}^{B}$	$n^{B}\left(k_{i}^{B}\right)$
0	2	$s\left(s-1\right){\left(s+1\right)}^{t-1}$	s−1	$s\left(s-1\right){\left(s+1\right)}^{t-1}$
1	2×2	$s\left(s-1\right){\left(s+1\right)}^{t-2}$	2(s−1)	$s\left(s-1\right){\left(s+1\right)}^{t-2}$
⋯	⋯	⋯	⋯	⋯
$t_{i}$	$2\times 2^{t_{i}}$	$s\left(s-1\right){\left(s+1\right)}^{t-t_{i}-1}$	$2^{t_{i}}\left(s-1\right)$	$s\left(s-1\right){\left(s+1\right)}^{t-t_{i}-1}$
⋯	⋯	⋯	⋯	⋯
t−1	$2\times 2^{t-1}$	$s\left(s-1\right){\left(s+1\right)}^{0}$	$2^{t-1}\left(s-1\right)$	$s\left(s-1\right){\left(s+1\right)}^{0}$
*t*	$2\times 2^{t}$	*s*	$2^{t}\left(s-1\right)$	*s*

**Table 2:** The local clustering coefficient of node in network $G_{s,r}^{B}\left(t\right)$.

*t*	$k_{i}^{B}$	$c(k_{i}^{B})$	$n^{B}\left(k_{i}^{B}\right)$
0	s−1	1	$s\left(s-1\right){\left(s+1\right)}^{t-1}$
1	2(s−1)	$\frac{s-2}{2(s-1)-1}$	$s\left(s-1\right){\left(s+1\right)}^{t-2}$
⋯	⋯	⋯	⋯
$t_{i}$	$2^{t_{i}}\left(s-1\right)$	$\frac{s-2}{2^{t_{i}}\left(s-1\right)-1}$	$s\left(s-1\right){\left(s+1\right)}^{t-t_{i}-1}$
⋯	⋯	⋯	⋯
t−1	$2^{t-1}\left(s-1\right)$	$\frac{s-2}{2^{t-1}\left(s-1\right)-1}$	$s\left(s-1\right){\left(s+1\right)}^{1}$
*t*	$2^{t}\left(s-1\right)$	$\frac{s-2}{2^{t}\left(s-1\right)-1}$	*s*

## Data Availability

Not applicable.

## Appendix A. The Values of *A*1–*A*6

In Appendix A, we give the analytical values of $A_{1},A_{2},A_{3},A_{4},A_{5},A_{6}$ involved in Theorem 4.

(A1) $$\begin{array}{cc} A_{1} & =\frac{s^{2}\left(s^{2}-1\right)+2{\left(s-1\right)}^{3}\left(s+1\right)-\left(3s^{2}+s\right){\left(s-1\right)}^{2}}{2s(s+1)},\\ A_{2} & =\frac{-2{\left(s-1\right)}^{3}-2s{\left(s-1\right)}^{3}+\left(3s^{2}+s\right){\left(s-1\right)}^{2}}{2s(s+1)},\\ A_{3} & =\frac{{\left(s-1\right)}^{3}}{s+1},\\ A_{4} & =\frac{s(s-1)}{2}-\frac{{\left(s-1\right)}^{2}\left(1-s^{2}r^{2}\right)}{s(1-r)(sr+1)}+\frac{\left[\left(s^{2}-s\right)r+s^{2}+s-2\right]{\left(s-1\right)}^{2}{\left(s+1\right)}^{2}}{2\left(1-r\right)[sr+1-{\left(s+1\right)}^{2}]\left(sr+1\right)},\\ A_{5} & =\frac{\left[\left(s^{2}-s\right)r+s^{2}+s-2\right]{\left(s-1\right)}^{2}}{2\left(1-r\right)[sr+1-{\left(s+1\right)}^{2}]},\\ A_{6} & =\frac{{\left(s-1\right)}^{2}\left(1-s^{2}r^{2}\right)}{s(1-r)(sr+1)}. \end{array}$$

## Appendix B. The Values of *B*1–*B*6

In Appendix B, we give the values of $B_{1},B_{2},B_{3},B_{4},B_{5},B_{6}$ involved in Theorem 5.

(A2) $$\begin{array}{cc} B_{1} & =\frac{4s^{2}-40s+24}{\left(s-4)(s+1\right)}-\frac{{\left(s-1\right)}^{2}}{s(1+s)}-\frac{4s^{2}-32s+16}{s^{2}-4s},\\ B_{2} & =\frac{4s^{2}-32s+16}{\left(s^{2}-4s\right)\left(1+s\right)}-\frac{{\left(s-1\right)}^{2}}{s(1+s)},\\ B_{3} & =-\frac{{\left(s-1\right)}^{2}}{1+s},\\ B_{4} & =\frac{4s^{2}-40s+24}{\left(s-4)(1+sr\right)}-\frac{\left(4s^{2}-32s+16\right)\left(1+s\right)}{s(s-4)(1+sr)}-\frac{{\left(s-1\right)}^{2}\left(s-2sr-1\right)\left(1+s\right)}{\left(1+s\right)\left(s^{2}r^{2}+sr\right)}\\ \; & \;\;\;-\frac{{\left(s-1\right)}^{2}\left(s-2sr-1\right)\left(1+s\right)}{\left(1+s\right)\left(s^{2}r^{2}+sr\right)\left(r-1\right)},\\ B_{5} & =\frac{4s^{2}-32s+16}{s(s-4)(1+sr)}+\frac{{\left(s-1\right)}^{2}\left(s-2sr-1\right)}{\left(1+s\right)\left(s^{2}r^{2}+sr\right)},\\ B_{6} & =\frac{{\left(s-1\right)}^{2}\left(s-2r-1\right)}{\left[(1+sr)(1+s)-s-1](r-1\right)}. \end{array}$$
